# *Chelidonium majus* L.: A Current Perspective on Isoquinoline Alkaloids, Emerging Phytochemicals, Alkaloid Biosynthesis, and Biological Activities

**DOI:** 10.3390/plants14172627

**Published:** 2025-08-23

**Authors:** Ramona Romanu, Sergio Liga, Maria Roberta Tripon, Florin Huiban, Dan Iliescu, Cristina Adriana Dehelean, Tulcan Camelia

**Affiliations:** 1 Doctoral School Engineering of Plant and Animal Resources, University of Life Sciences “King Mihai I” from Timișoara, Aradului Street No. 119, 300645 Timisoara, Romania; romanuram@yahoo.com (R.R.); roberta.tripon@usvt.ro (M.R.T.); florin.huiban@usvt.ro (F.H.); 2 Research Center for Pharmaco-Toxicological Evaluation, “Victor Babeș” University of Medicine and Pharmacy Timisoara, Eftimie Murgu Square No. 2, 300041 Timișoara, Romania; cadehelean@umft.ro; 3 Faculty of Chemical Engineering, Biotechnologies and Environmental Protection, Politehnica University Timișoara, Vasile Pârvan No. 6, 300223 Timișoara, Romania; sergio.liga96@gmail.com; 4 Faculty of Engineering and Applied Technologies, University of Life Sciences “King Mihai I” from Timișoara, Calea Aradului No. 119, 300645 Timișoara, Romania; 5 Research Institute for Biosecurity and Bioengineering, University of Life Sciences “King Mihai I” from Timisoara, 300645 Timișoara, Romania; 6 Faculty of Medicine, “Victor Babeș” University of Timișoara, 2 Eftimie Murgu Square, 300041 Timisoara, Romania; 7 University Clinic of Toxicology, Drug Industry, Management and Legislation, Faculty of Pharmacy, “Victor Babeș” University of Medicine and Pharmacy Timișoara, Eftimie Murgu Square No. 2, 300041 Timișoara, Romania

**Keywords:** *Chelidonium majus*, greater celandine, bibliometric analysis, isoquinoline alkaloids, lignanamides, therapeutic potential

## Abstract

Phytochemistry serves as a vital bridge between traditional medicinal knowledge and modern scientific research, with important implications for pharmaceutical and industrial applications. This review offers an updated and integrated perspective on *Chelidonium majus* (greater celandine), focusing on its isoquinoline alkaloids—the principal bioactive constituents—alongside emerging phytochemicals (e.g., lignanamides, polyphenols). Detailed biosynthetic pathways of isoquinoline alkaloids are described, tracing their formation from the shikimate pathway to multiple structural subclasses. Reported biological activities include anticancer, antioxidant, anti-inflammatory, antimicrobial, antiviral, and immunomodulatory effects. A bibliometric analysis was conducted using VOSviewer software (Scopus dataset, 2015–2025), enabling the identification of major research themes and temporal trends. These research tools supported a structured and data-driven overview of the current scientific landscape. However, additional studies are needed to optimize its therapeutic use while ensuring efficacy and safety.

## 1. Introduction

Modern drug discovery is increasingly integrating traditional knowledge with cutting-edge scientific methodologies to identify and optimize plant-based therapeutics, due to advances in analytical techniques, molecular biology, and computational approaches [1,2,3].

Nowadays, herbal therapy is widely recognized as an alternative and adjuvant treatment method for various acute and chronic diseases, alongside the use of plant-derived drugs [4]. Among the many medicinal herbs, greater celandine stands out due to its diverse bioactive profile and historical use in both traditional and modern herbal medicine.

Based on APG IV, the greater celandine belongs to the *Papaveraceae* family, which is part of the Ranunculales order within the eudicot group of vascular plants. This species is the only member of the *Chelidonium* genus. It has a wide distribution across temperate regions in Europe and Asia. It is endemic to Europe and Western Asia, but it has been introduced to North America, where it has become naturalized in numerous areas. Central and eastern Europe is where it is most prevalent, but it can be found as far east as China and Korea. In North America, it is frequently regarded as an invasive species. The plant thrives in habitats that are moist and shaded, such as woodland edges, hedgerows, and disturbed areas [5,6].

**Figure 1:**
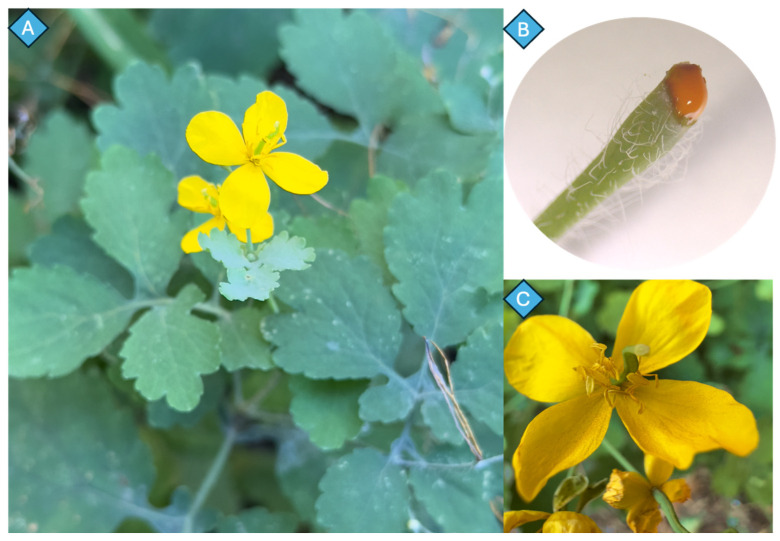
(**A**–**C**) A photograph of *C. majus* plant.

*Chelidonium majus* L. (Papaveraceae) is an herbaceous perennial that can reach heights of 30–100 cm, with a branched, erect, and slightly pubescent stem that, when broken, exudes a distinctive yellow-orange latex rich in isoquinoline alkaloids, traditionally used in folk medicine for the topical treatment of warts, eczema, fungal infections, and other skin disorders (Figure 1A,B) [7].

The leaves have deep lobes and are pinnately divided, resulting in a fern-like appearance. Their upper surface is bluish-green and paler underneath, with fine hairs often covering them (Figure 1B). The leaves are arranged in an alternating pattern on the stem, with the basal leaves being bigger and petiolate and the upper leaves being smaller and more sessile. The flowers are bright yellow, have an approximate diameter of 1.5–2.5 cm, and have four rounded petals, two falling off sepals, and numerous stamens (Figure 1C). The arrangement of these flowers is umbel-like inflorescences, with inflorescences located at the tips of the stems. The fruit’s capsule, a dehiscent silique, which is 3–5 cm long and has two valves, splits open to release numerous small, black seeds [8,9,10]. Elaiosomes found in these seeds attract ants and help in seed dispersion through myrmecochory (ant-mediated dispersion) [11].

Ecologically, *C. majus* thrives in semi-shaded habitats, often along hedgerows, woodland edges, disturbed soils, and moist wastelands [12]. In traditional medicine, the plant has a long ethnopharmacological history dating back to Greco-Roman times, when it was used for treating warts, skin diseases, and eye conditions. In European folk medicine, fresh latex was applied topically for wart removal, while aerial parts and roots were prepared as infusions or tinctures for liver and gallbladder ailments, digestive disorders, and as a mild sedative [13,14,15].

Despite greater celandine’s long-standing application in folk medicine, accumulating clinical evidence has raised concerns regarding its safety profile, particularly its hepatotoxic potential [16,17,18]. These adverse reactions are believed to result from idiosyncratic mechanisms, possibly involving oxidative stress, mitochondrial dysfunction, and immune-mediated responses triggered by the greater celandine’s alkaloid constituents. Regulatory committees in several European countries have already issued safety warnings or imposed restrictions on the use of greater celandine, particularly in products intended for long-term or unsupervised use [19].

**Figure 2:**
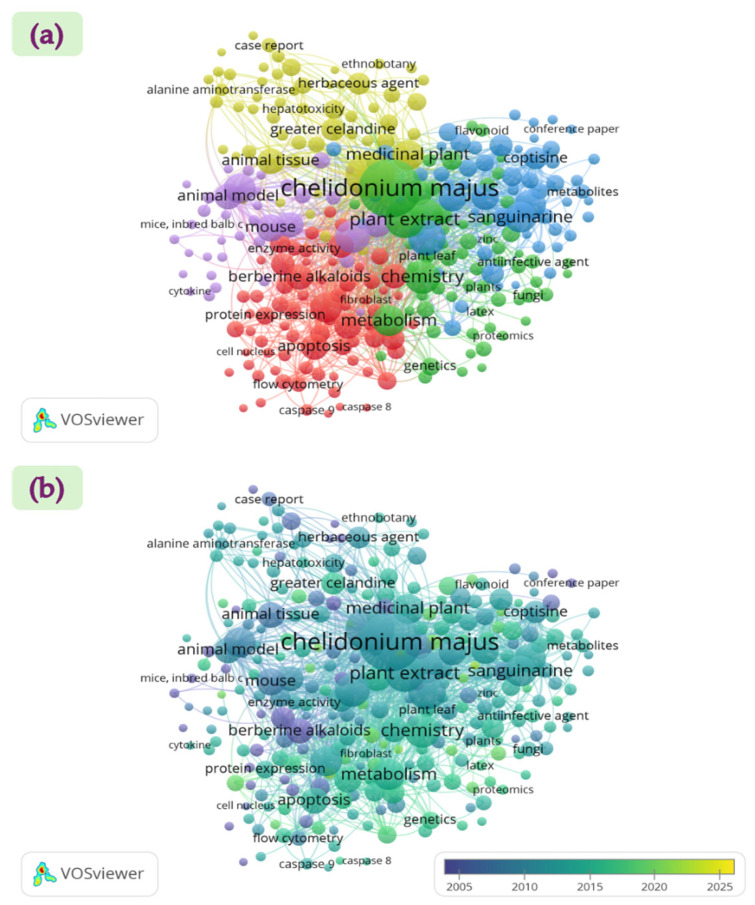
Keyword co-occurrence analysis of publications on greater celandine retrieved from the Scopus database (*n* = 1518): (**a**) Network visualization showing relationships between terms (curved lines) and frequency of occurrence (circle size); and (**b**) Overlay visualization indicating relatively limited research activity during the 2015–2025 period.

A keyword co-occurrence analysis was conducted to identify thematic structures and research trends in scientific publications related to *C. majus*. Terms were extracted from the titles, abstracts, and author keywords of all selected documents. The visualization maps (Figure 2) were generated in VOSviewer software (Version 1.6.20) [20], from a Scopus dataset comprising 1518 publications. In the network visualization (Figure 2a), curved lines represent relationships between terms, and circle size reflects the frequency of keyword occurrence. A minimum occurrence threshold of five was applied, resulting in five distinct keyword clusters. The overlay visualization (Figure 2b) indicates that research activity on greater celandine has been relatively limited during the 2015–2025 period.

This review addresses these developments by (i) integrating well-known isoquinoline alkaloids with emerging classes such as lignanamides and polyphenols; (ii) presenting the most detailed, step-by-step account of alkaloid biosynthesis in *C. majus* to date; (iii) conducting a bibliometric co-occurrence analysis of publications (2005–2025 period) using VOSviewer to identify thematic clusters and research trends; and (iv) embedding phytochemical and bioactivity data findings within a broader scientific context.

## 2. Emerging Phytochemicals and Biological Functions

**Figure 3:**
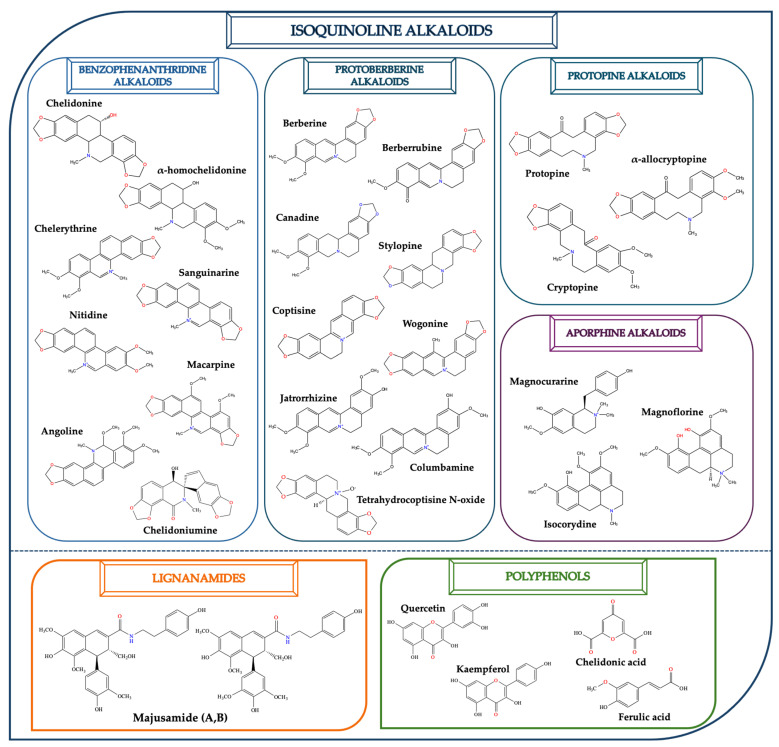
The main active biologically constituents of *C. majus*.

*C. majus* possesses a complex chemical profile, with isoquinoline alkaloids representing the principal biologically active constituents. A concise overview of these phytochemicals is presented below (Figure 3).

### 2.1. Isoquinoline Alkaloids

In greater celandine, four major structural groups of isoquinoline-derived alkaloids have been reported: (i) phenanthridine or 3,4-benzophenanthridine alkaloids (e.g., chelidonine, chelerythrine, sanguinarine, chelidoniumine, nitidine, macarpine, angoline, and their derivatives); (ii) protoberberine alkaloids (e.g., berberine, berberrubine, canadine, stylopine, wogonine, coptisine, jatrorrhizine, columbamine, tetrahydrocoptisine N-oxide); (iii) protopine alkaloids (e.g., protopine, cryptopine); and (iv) aporphine alkaloids (e.g., magnocurarine, magnoflorine, isocorydine) [21,22].

### 2.2. Lignanamides

In addition to the well-studied isoquinoline alkaloids previously described, *C. majus* has also been shown to contain a variety of other bioactive compound classes, such as lignanamides. Lignanamides, a distinct class of secondary metabolites characterized by the presence of amide functional groups within lignan frameworks, also referred to as bis-alkaloids, are presumed to arise via peroxide-mediated coupling reactions of phenylpropanoid amide monomers or dimers [23]. A comprehensive phytochemical study by Huang et al. led to the isolation of two novel lignanamides (majusamides A and B), as well as two new alkaloids (chelidoniumine and tetrahydrocoptisine N-oxide), from a 75% ethanol extract of the aerial parts of the greater celandine. Pharmacological testing revealed that several of these compounds exhibited moderate inhibitory activity against nitric oxide production in lipopolysaccharide (LPS)-stimulated BV-2 macrophage cells, suggesting notable anti-inflammatory potential [24].

### 2.3. Polyphenols and Other Phytochemicals

In addition to these isoquinoline alkaloids and lignanamides, greater celandine also contains other different constituents, such as polyphenols (e.g., chelidonic acid, ferulic acid, caffeic acid, quercetin, kaempherol, rutoside) [25,26], phytosterols (e.g., ergosterol), biogenic amines (e.g., histamine, tyramine), and non-essential and essential amino acids (e.g., tyrosine, alanine, serin, isoleucine, valine, threonine) [13,21,22].

It has also been reported to accumulate a wide range of macro- and microelements, including calcium, potassium, magnesium, sodium, iron, manganese, copper, zinc, lead, cadmium, chromium, and nickel, which reflect both its physiological requirements and its ability to absorb elements from the surrounding urban environment [12,27,28].

The following table will briefly introduce some emergent phytochemicals from greater celandine, which have significant biological functions (Table 1).

## 3. Biosynthesis of Isoquinoline Alkaloids

**Figure 4:**
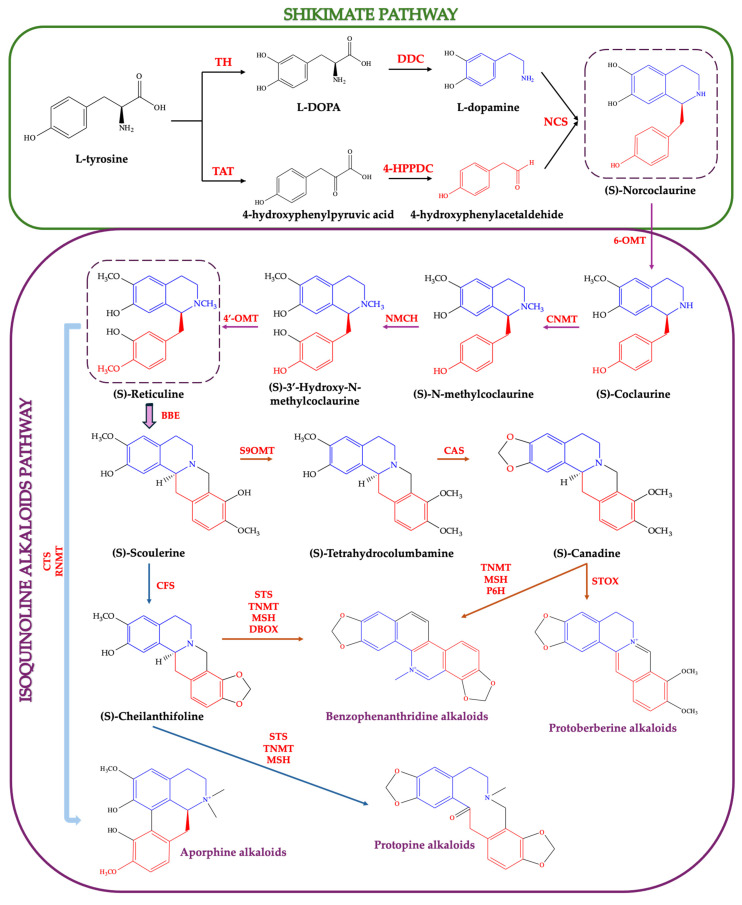
Overview of the main steps of the isoquinoline alkaloids biosynthesis pathway [87,88,89,90,91,92,93].

Isoquinoline alkaloids constitute a large and structurally diverse class of nitrogen-containing secondary metabolites, predominantly found in plants. Greater celandine is particularly rich in these compounds, which represent its principal biologically active constituents. From a chemical point of view, these alkaloids are derived from the shikimate pathway, and characterized by a benzylisoquinoline skeleton, which consists of a benzyl group (−CH_2_C_6_H_5_) attached to an isoquinoline core. In one route, L-tyrosine is hydroxylated by tyrosine hydroxylase (TH) to L-DOPA and then decarboxylated by DOPA decarboxylase (DDC) to form dopamine. In the other route, tyrosine undergoes transamination by tyrosine aminotransferase (TAT) to 4-hydroxyphenylpyruvic acid, followed by decarboxylation by 4-hydroxyphenylpyruvate decarboxylase (4-HPPDC) to yield 4-hydroxyphenylacetaldehyde. These two intermediates are then condensed by norcoclaurine synthase (NCS) to form (S)-norcoclaurine, the core scaffold for isoquinoline alkaloid molecules biosynthesis (Figure 4) [87,88,89,90,91,92,93]. Subsequent enzyme modifications, including methylation, oxidation, hydroxylation, and rearrangements, generate a vast array of benzylisoquinoline derivatives [88].

Between the shikimate and isoquinoline alkaloid biosynthetic pathways lies an intermediate sequence in which (S)-norcoclaurine undergoes successive methylation and hydroxylation steps to yield (S)-reticuline [89,90,93]. Initially, (S)-norcoclaurine is methylated at the 6-hydroxyl position by norcoclaurine 6-O-methyltransferase (6-OMT) to produce (S)-coclaurine. This is followed by N-methylation via coclaurine N-methyltransferase (CNMT), generating (S)-N-methylcoclaurine. Subsequent hydroxylation at the 3′ position by the cytochrome P450-dependent (S)-N-methylcoclaurine 3′-hydroxylase (NMCH) produces (S)-3′-hydroxy-N-methylcoclaurine, which is then methylated at the 4′-hydroxyl group by 3′-hydroxy-N-methylcoclaurine 4′-O-methyltransferase (4′-OMT) to form (S)-reticuline. The committed step in isoquinoline alkaloid biosynthesis (e.g., benzophenanthridine, protoberberine, protopine alkaloids) is the stereoselective conversion of (S)-reticuline to (S)-scoulerine, catalyzed by the berberine bridge enzyme (BBE) [93].

In the protoberberine and benzophenanthridine biosynthetic pathways, (S)-scoulerine undergoes O-methylation by scoulerine 9-O-methyltransferase (S9OMT) to produce (S)-tetrahydrocolumbamine. This intermediate is subsequently converted to (S)-canadine via methylenedioxy bridge formation catalyzed by canadine synthase (CAS). (S)-Canadine serves as a central precursor for multiple downstream alkaloid classes, including benzophenanthridines and protoberberines, through a series of oxidative and methylation reactions mediated by enzymes (e.g., TNMT, tetrahydroprotoberberine-cis-N-methyltransferase; MSH, N-methylstylopine-14-hydroxylase; P6H, protopine-6-hydroxylase; STOX, (S)-tetrahydroprotoberberine oxidase) [87,88,89,90,91,92,93].

In another branch of the biosynthetic pathway, (S)-scoulerine is converted to (S)-cheilanthifoline via methylenedioxy bridge formation catalyzed by cheilanthifoline synthase (CFS). The introduction of a second methylenedioxy bridge, followed by oxidative rearrangement catalyzed by a series of enzymes including stylopine synthase (STS), tetrahydroprotoberberine-N-methyltransferase (TNMT), and methylstylopine hydroxylase (MSH), leads to the formation of protopine. Protopine serves as a direct precursor to various protopine-derived alkaloids, and to benzophenanthridine alkaloids (e.g., sanguinarine and chelerythrine) via the sequential action of dihydrobenzophenanthridine oxidase (DBOX) [90,91,92,93].

(S)-Reticuline also functions as a key precursor in the biosynthesis of aporphine alkaloids. In this branch, (S)-reticuline undergoes oxidative phenol coupling catalyzed by corytuberine synthase (CTS) to produce (S)-corytuberine. Subsequent methylation and oxidation reactions, mediated by reticuline N-methyltransferase (RNMT) and related enzymes, yield a variety of aporphine-type alkaloids, including magnoflorine and isocorydine [87,88,89,90,91,92,93]. These compounds are characterized by a tetracyclic aporphine skeleton derived from the intramolecular coupling of the benzyl and isoquinoline moieties of reticuline.

## 4. Recent Data on the Bioactivity of *Chelidonium majus*

Greater celandine has been used in traditional medicine since ancient times. Celandine was thought by Dioscorides to have the most potent medicinal properties in treating visual disorders, eye diseases, and relieving dental inflammation in the first century [94,95]. Today, celandine is known to possess a range of therapeutic benefits, primarily because of its complex phytochemistry, but it also has some toxicological risks [13,18,22,96]. Below, we will outline the most recent data concerning the biological actions of greater celandine.

### 4.1. Anticancer Activity

Using multiple hyphenated instrumental techniques including HPLC-DAD, Petruczynik et al. investigate the anticancer activity and measure specific isoquinoline alkaloids in extracts from *C. majus*. They evaluated the cytotoxic effects of this extract on human cancer cell lines: (i) pharyngeal squamous carcinoma (FaDu), (ii) tongue squamous carcinoma (SCC-25), (iii) breast adenocarcinoma (MCF-7), and (iv) triple-negative breast adenocarcinoma (MDA-MB-231). According to the results, all of the tested extracts demonstrated significant cytotoxicity against these cancer cell lines. Notably, the root extract of *C. majus* showed the highest cytotoxic activity against FaDu and MDA-MB-231 cells, with IC_50_ values of 2.52 ± 0.25 µg/mL and 2.61 ± 0.33 µg/mL, respectively. These findings indicate that the celandine extract studied has potent anticancer properties, which require further in vivo studies to investigate its potential as therapeutic agents [97].

Tuzimsky et al. investigated the chemical composition and anticancer properties of extracts from *C. majus*, *Mahonia aquifolium* and *Sanguinaria canadensis*, focusing on their isoquinoline alkaloid content and cytotoxic effects on various human cancer cell lines (e.g., FaDu, SCC-25, MCF-7, MDA-MB-231). The researchers used liquid chromatography to identify and quantify several isoquinoline alkaloids in the plant extracts, with a particular focus on celandine, such as chelidonine, sanguinarine, chelerythrine, and berberine. The results revealed that this time the root extracts showed significant cytotoxicity, particularly against melanoma cell lines. The root extract had an IC_50_ of 12.65 µg/mL against A375 cells and 1.93 µg/mL against SK-MEL-3 cells. Furthermore, the celandine root extract was tested on a *Danio rerio* (zebrafish) larvae xenograft model and demonstrated a significant reduction in the number of cancer cells in vivo, confirming its potent anticancer activity. According to these findings, the celandine extract is a potential candidate for further research and development as natural anticarcinogenic agents [98].

In another study, Nile et al. evaluate the cytotoxic effect against the HeLa and CaSki human cervical cancerous cells, using extracts made from different celandine parts (e.g., leaf, stem, flower, pod, and root). According to their findings, the pod and flower extracts exhibited the most potent cytotoxic effects after 48 h of exposure at 1000 μg/mL, with the pod extracts showing an IC_50_ value of 6.1% (HeLa) and 0.72% (CaSki), while the flower extracts showed an IC_50_ value of 7.9% (HeLa) and 9.5% (CaSki), respectively [99]. In another example, Nawrot et al. investigated whether *C. majus* latex’s proteins and alkaloids had a combined effect on human cervical carcinoma cells. A novel major uncharacterized nucleic acid binding latex protein, CmMLP1, which contains 147 amino acids, was isolated by them, and they discovered its association with alkaloids like berberine, 8-hydroxycheleritrine, and dihydroberberine. Using molecular docking analyses, it was found that CmMLP1 has a hydrophobic cavity that has a high affinity for these alkaloids. In vitro cytotoxicity assays demonstrated that fractions containing both CmMLP1 and the associated alkaloids significantly reduced the viability of human cervical cancer cell lines, including both HPV-positive (HeLa) and HPV-negative (C33A) cells. It was observed that the combination had a stronger cytotoxic effect on HPV-positive cells, which suggests a potential synergistic interaction between CmMLP1 and the alkaloids. These findings shed light on the molecular interactions within C. majus latex and its potential use in cervical cancer treatment [100].

Terzic et al. investigated the cytotoxic effect of three extracts obtained from greater celandine with different solvents, such as ethyl acetate, methanol, and water. The effect of *C. majus* extracts on cancer cell viability was assessed across a range of cancer cell lines, including HGC-27 (gastric cancer), DU-145 (prostate carcinoma), HT-29 (colon adenocarcinoma), MDA-MB-231 (breast adenocarcinoma), HELA (cervix adenocarcinoma), and A549 (lung cancer), and normal cell line HEK-293 (kidney epithelia). The ethyl extract demonstrated favorable IC_50_ values across multiple cancer cell lines, including MDA-MB-231, HGC-27, HT-29, and DU, with IC_50_ values of 8.108 μg/mL, 8.27 μg/mL, 9.078 μg/mL, and 22.1 μg/mL, respectively. The methanolic extract showed efficacy against HT, HGC-27, and HELA with IC_50_ values of 4.525 μg/mL, 8.538 μg/mL, and 18.55 μg/mL, respectively. The water extract, on the other hand, generally displayed less favorable IC_50_ values, which suggests a weaker inhibitory effect [101].

Natanzi et al. evaluated the impact of alkaloids extracted from *C. majus* on the Daudi Burkitt lymphoma cell line. Their study focused on assessing cytotoxicity, apoptosis induction, and the expression of the vascular endothelial growth factor (VEGF) gene, a key regulator of angiogenesis in cancer progression. The results revealed that the alkaloid extract exhibited dose-dependent cytotoxicity against Daudi cells, with a 50% cytotoxic concentration (CC_50_) determined to be 56.35 µg/mL after 48 h. According to flow cytometry analysis, CC_50_ concentration treatment caused 1.4% early apoptosis and 37.94% late apoptosis in Daudi cells. VEGF gene expression was downregulated in Daudi cells treated with the alkaloid extract in dose-dependent manner, as demonstrated by real-time PCR results. Their study results provide a foundation for further in vivo studies to explore the therapeutic potential of *C. majus* alkaloids as natural anticancer agents [102].

In a study conducted by Salehi et al., the anticancer potential of isoquinoline alkaloids extracted from *C. majus* was investigated, with a specific focus on their ability to bind G-quadruplex (G4) DNA structures. Among the screened compounds, berberine and chelerythrine emerged as the most potent G4 binders, showing high affinity for telomeric and oncogene–promoter G-quadruplexes. These compounds demonstrated significant stabilization of G4 structures, suggesting their potential as transcriptional repressors of cancer-associated genes. In vitro cytotoxicity assays revealed that both compounds significantly reduced cell viability in MCF-7 (breast cancer) and HCT116 (colorectal cancer) cell lines, with IC_50_ values ranging from 2.5 to 5.0 μM, depending on the cell type and compound [103].

### 4.2. Anti-Inflammatory and Analgesic Activities

Mikołajczak et al. studied the analgesic and anti-inflammatory effects of *C. majus* herb extracts by evaluating three fractions: full water extract (FWE), protein-enriched fraction (PEF), and non-protein fraction (NPF). In the “hot plate” analgesia test, FWE and PEF administered at a dose of 10 mg/kg (intraperitoneally) exhibited significant analgesic activity three hours post-administration, with responses comparable to morphine (5 mg/kg) (*p* < 0.05). Interestingly, FWE also induced a pro-inflammatory effect at the same time point, as indicated by elevated cytokine levels. Furthermore, all three fractions significantly reduced peripheral IL-1 and IL-4 levels, suggesting systemic anti-inflammatory effects [104].

Zielinska et al. investigated the effects of *C. majus* root extract and its alkaloids (e.g., coptisine, berberine, chelidonine, chelerythrine, and sanguinarine) on cytokine secretion in lipopolysaccharide-stimulated human neutrophils. Berberine, chelidonine, and chelerythrine significantly reduced TNF-α secretion in a concentration-dependent manner, particularly at 10 µM, with chelidonine and chelerythrine exhibiting minimal cytotoxicity at this level. Sanguinarine was identified as the most potent inhibitor of IL-1β production, showing significant effects at 1 µM. However, it also induced an increase in IL-8 and TNF-α secretion at lower concentrations (e.g., 0.1 µM) and displayed high cytotoxicity at concentrations ≥ 5 µM. These results suggest selective, dose-dependent immunomodulatory effects of the individual alkaloids, with differing safety profiles [105].

In in vitro studies, chelerythrine extracted from *C. majus* significantly suppressed the production of key inflammatory mediators, such as nitric oxide (NO), tumor necrosis factor-alpha (TNF-α), interleukin-6 (IL-6), and IL-1β in LPS-stimulated RAW264.7 macrophages. It also downregulated the expression of inducible nitric oxide synthase (iNOS) and cyclooxygenase-2 (COX-2) in a dose-dependent manner, without inducing cytotoxicity at concentrations ≤ 10 µM. Functionally, it inhibited GAPDH enzymatic activity and decreased pyruvate levels, indicating suppression of glycolytic flux [106].

### 4.3. Antiviral Activity

Musidlak et al. provides an in-depth analysis of how different components of *C. majus* latex (e.g., specifically its crude form, protein fraction, alkaloid-rich fraction) interact with the human papillomavirus (HPV) replication cycle. Their results showed that crude latex exhibited the most antiviral effect, decreasing infectivity by approximately 72.5%. The protein fraction and alkaloid-rich fraction reduced infectivity by 35.6% and 22.9%, respectively. Increased TNF-α secretion was associated with the protein fraction of the latex, which suggests a potential for inducing inflammatory responses. In contrast, the latex components did not impact the attachment of HPV pseudovirions to host cells or the degradation of viral capsid proteins (L1 and L2), suggesting that their antiviral activity does not relate to the initial stages of infection. Both alkaloids and proteins found in *C. majus* latex are responsible for its antiviral properties against HPV, as evidenced by the study. The viral replication cycle is impeded by these components at post-entry but before nuclear translocation stages without affecting viral attachment or capsid integrity [107].

In their observational case series, Gardin and Braga evaluated 20 outpatients diagnosed with COVID-19 who were treated with a 10% mother tincture of *C. majus* L. The tincture was administered orally at a dose of 20–30 drops, three times daily, over a treatment period ranging from 3 to 12 days. The patient cohort included individuals aged 14–71 years, and the initiation of treatment occurred between 1 and 19 days after the onset of symptoms. Notably, complete or near-complete symptom resolution was observed within 1–9 days of initiating treatment. Although the observational nature and small sample size of their study limit its generalizability, the study suggests that *C. majus* may contribute to symptom alleviation in mild COVID-19 cases [108].

### 4.4. Antimicrobial and Antifungal Activities

The study of Hong et al. investigated the antioxidant and antimicrobial activities of *C. majus* L. against oral microorganisms. According to the DPPH and ABTS radical scavenging assay, the extract demonstrated significant antioxidant activity and the most significant levels of phenolic and flavonoid content (212.00 mg CAE/g, 135.70 mg NE/g). Furthermore, the antimicrobial assay demonstrated strong antimicrobial activity against oral microorganisms (e.g., *S. mutans*, *S. gordonii*, *S. sobrinus*, *S. sanguinis*), and it was found that the most inhibitive is the ethyl acetate fraction [81].

Qi et al. investigated the alkaloid impact of methicillin-resistant *Staphylococcus aureus* (MRSA) and methicillin-sensitive *Staphylococcus aureus* (MSSA) on the motility, longevity, and reactive oxygen species levels of wild-type worms (N2 strain). The results indicated that the concentration of total alkaloids, which was between 0.625 – 10 mg/mL, had a significant effect on MRSA. Increased concentration correlates with an increase in antibacterial activity, suggesting that total alkaloids exert antimicrobial effects at specific doses [109]. In another study conducted by Krzyzek et al., they investigated the antimicrobial efficacy of plant extracts (*C. majus* and *Corydalis cheilanthifolia*) against a multidrug-resistant strain of *Helicobacter pylori*. The findings revealed that the *C. majus* extract shows impressive antibacterial properties, with an MIC of 128 µg/mL and an MBC of 256 µg/mL, respectively [110].

In the study by Lu et al., the nematicidal activities of two isoquinoline alkaloids—berberine and sanguinarine—isolated from C. majus were assessed against the root-knot nematode *Meloidogyne incognita*. In vitro assays demonstrated strong dose-dependent lethality, with both compounds significantly inhibiting second-stage juvenile (J2) viability and egg hatching. At concentrations of 100 μg/mL, berberine and sanguinarine achieved over 90% J2 mortality within 48 h [111].

In a study conducted by Sadecki et al., aerial tissues of *C. majus* were investigated for the presence of antimicrobial peptides (AMPs). Using a PepSAVI-MS pipeline, the team identified a novel, cysteine-rich peptide, CM-AMP1. The synthetic peptide exhibited potent membrane-lytic activity, achieving near-complete inhibition of *E. coli* ATCC 25922 and MRSA USA300 at micromolar concentrations [112].

### 4.5. Antioxidant Activity

Segneanu et al. investigated the development of a novel carrier system that combines extracts from Romanian wild-grown greater celandine with gold nanoparticles. The extract–nanoparticle system exhibited more antioxidant activity, resulting in a 3.8% increase in flavonoid content and a 24.6% increase in FRAP results, compared to the herb extract alone [113]. Khodabande et al. evaluated greater celandine leaf extracts across three distinct phenological stages (e.g., vegetative, flowering, fruiting) indicating that the plant’s biochemical composition and antioxidant potential vary significantly with developmental phase. The flowering stage was identified as the most phytochemically active, exhibiting the highest concentrations of total phenols (17.8 ± 1.59 mg/g DW) and total flavonoids (69.7 ± 0.86 mg/g DW). These compounds are known for their strong free radical scavenging properties, suggesting that extracts collected during this period may offer the greatest therapeutic benefit. The vegetative stage demonstrated the highest carotenoid content (2.083 mg/g DW) and protein content (0.27 ± 0.034 mg/g DW), indicating intense metabolic and structural activity during early growth. The growth stage (vegetative to early flowering) also showed the maximum DPPH radical scavenging activity at 408.88 ± 24.83 g/g DW, reflecting a potent ability to neutralize reactive oxygen species (ROS). Conversely, the FRAP assay revealed that ferric-reducing antioxidant power peaked during the fruiting stage (1.75 ± 0.04 mg/g FW), possibly due to the accumulation of secondary metabolites necessary for reproductive tissue protection. Additionally, the soluble sugar content reached its maximum during flowering (0.338 ± 0.009 mg/g DW), supporting the energetic requirements of the plant during this intensive phase of development. These findings highlight the critical role of harvest timing in optimizing the bioactivity of *C. majus* extracts [114].

In a study conducted by Dostemessova et al., the antioxidant properties of *C. majus* were systematically evaluated using methanolic extracts obtained from both aerial and root parts of plants collected at three distinct ecological sites in the Kungei-Alatau region. The study demonstrated that root extracts exhibited significantly higher antioxidant activity compared to aerial parts. Notably, the Kolsay (N1) root sample showed the most pronounced antioxidant potential, with DPPH (1735.85 ± 14.28 µmol TE/mL), ABTS (917.97 ± 18.06 µmol TE/mL), and PFRAP (18.93 ± 0.24 mmol AAE/mL) values. Additionally, the highest total phenolic content (2617.62 ± 9.59 µg GAE/mL) was observed in the root extract from the Kayindy (N2) site. The findings suggest that the antioxidant activity of greater celandine is primarily linked to its phenolic content rather than alkaloid concentration. The authors also emphasized that environmental conditions, such as soil pH and composition, play a critical role in modulating the phytochemical profile of the plant [115].

### 4.6. Immunomodulatory Activity

Zielinska et al. carried out a study to examine how greater celandine extracts and their individual alkaloids impact the production of cytokines in human neutrophils stimulated with lipopolysaccharide. In the root extract of celandine, 25 compounds were found, with chelerythrine being the most abundant (1761.22 µg/g), followed by sanguinarine (1373.80 µg/g), chelidonine (1181.41 µg/g), and coptisine (1077.06 µg/g). Furthermore, five individual alkaloids (e.g., coptisine, berberine, chelidonine, chelerythrine, sanguinarine) were evaluated on the secretion of pro-inflammatory cytokines IL-1β, IL-8, and TNF-α. The results showed that celandine root extract itself increased the secretion of all three cytokines in a concentration-dependent manner (1.25–12.5 µg/mL). The study concluded that while alkaloids from celandine (e.g., chelidonine, chelerythrine) can regulate cytokine production, other alkaloids (e.g., coptisine and sanguinarine) have significant cytotoxic effects, which limits their therapeutic applications [105].

### 4.7. Insecticidal Activity

In a study conducted by Zou et al., greater celandine was shown to exhibit potent insecticidal activity against *Lymantria dispar* larvae, primarily due to its rich content of isoquinoline alkaloids. Both the crude extract (CECm) and the total alkaloid fraction (TACm) demonstrated significant larvicidal effects, with coptisine identified as the most abundant compound. Treatment with TACm significantly reduced larval food intake, growth rate, and assimilation efficiency, indicating pronounced antifeedant and growth-inhibitory effects. Enzymatic assays revealed that CECm and TACm strongly inhibited the activity of key detoxification and antioxidant enzymes (e.g., acetylcholinesterase (AChE), carboxylesterase (CarE), glutathione S-transferase (GST), superoxide dismutase (SOD), catalase (CAT)) in both in vitro and in vivo experiments [116].

Given the diversity of experimental approaches and outcomes reported, a comprehensive summary table was added to consolidate key information on plant part, extraction method, and biological activity (Table 2).

## 5. Conclusions and Future Perspectives

*Chelidonium majus* L. remains a phytochemically rich species of high pharmacological interest, characterized by a predominance of isoquinoline alkaloids and complemented by emerging classes of bioactive constituents such as lignanamides and polyphenols. Advances in analytical chemistry have refined our understanding of its complex alkaloid biosynthetic pathways, from shikimate precursors through multiple structural subclasses, including protoberberine, benzophenanthridine, aporphine, and protopine alkaloids.

Biological studies consistently support the plant’s anticancer, antioxidant, anti-inflammatory, antimicrobial, antiviral, and immunomodulatory activities, yet the translation of these effects into clinical practice is hindered by variability in chemical composition, gaps in pharmacokinetic data, and unresolved safety concerns, particularly regarding hepatotoxicity. The balance between therapeutic potential and toxic risk remains a critical research priority, and further controlled clinical trials and mechanistic toxicology studies are essential to delineate its therapeutic window and establish evidence-based safety guidelines.

## Figures and Tables

**Table 1:** Biological functions of some emerging phytochemicals isolated from *C. majus*.

Phytochemical Group	Phytoconstituent	Biological Functions	References
Benzophenanthridines	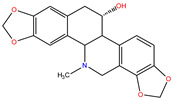 **Chelidonine**	▪ Anti-inflammatory activity: suppress the IL-1β-mediated catabolism and inflammation of chondrocytes, and the NF-kB pathway activation ▪ Anticancer activity: induce G2/M cell cycle arrest by downregulating CDK1, S cell cycle arrest by upregulating p21 and p53 with GADD45a increase, and finally triggered apoptotic cell death with caspase-3 cleavage in BxPC-3 and MIA PaCa-2 cells; regulate EGFR-AMPK signaling pathway, and reduce EGFR phosphorylation; exhibit a pronounced inhibitory effect on the proliferation of B16F10 cells	[29,30,31,32]
	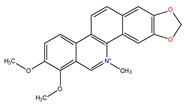 **Chelerythrine**	▪ Anti-inflammatory activity: inhibitory effects on the GluN2A and GluN2B NMDAR receptor in primary cultured cortical neurons ▪ Antiviral activity: potent inhibitor of ZIKV infection that targets the ZIKV NS4B protein; inhibitory activity suitable for cerebral vasospasm prevention and eryptosis reduction, as well as beneficial effects in suppressing pulmonary inflammation (e.g., SARS-CoV-2) ▪ Antifungal activity: inhibition on biofilm formation of the pathogens (e.g., *Candida albicans* and *Cryptococcus neoformans*) ▪ Anti-tumor activity against renal cells carcinoma is mainly induced by activation of the ER stress pathway and inhibition of STAT3 phosphorylation ▪ Affinity-labeling inactivator of CYP3A4 ▪ Inhibits Aβ aggregation, and induces Aβ disaggregation in Alzheimer’s disease	[33,34,35,36,37,38,39]
	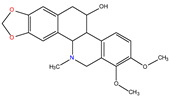 **α-homochelidonine**	▪ Antiproliferative activity	[40]
	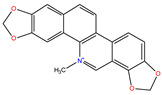 **Sanguinarine**	▪ Antimicrobial activity: effectively potentiated aminoglycoside killing on diverse bacterial pathogens (e.g., *Escherichia coli*, *Acinetobacter baumannii*, *Klebsiella pneumonia* and *Pseudomonas aeruginosa*) ▪ Agonist of TRPA1 channel ▪ Anti-inflammatory activity ▪ Antiplatelet and antithrombotic activities: reduce phosphorylation of the downstream signaling pathways in collagen specific receptor GPVI; inhibit integrin αIIbβ3 outside-in signaling via reducing β3 and Src (Tyr-416) phosphorylation ▪ Anticancer activity	[41,42,43,44,45,46]
	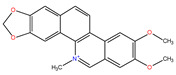 **Nitidine**	▪ Anticancer activity ▪ Anti-inflammatory activity: inhibit fibroblast-like synoviocytes-mediated rheumatoid synovial inflammation and joint destruction; therapeutic potential for rheumatoid arthritis ▪ Neuroprotective activity	[47,48,49,50,51]
Protoberberines	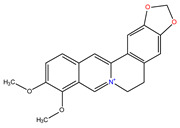 **Berberine**	▪ Anticancer activity: arrests human cancer cells in the G1 and G2/M phases; significantly increase the expression of caspase genes CASP3, CASP8 and CASP9, and proapoptotic genes BAK1, BAX and BIK ▪ Anti-inflammatory activity: reduce prostaglandin E2 (PGE2) production, and inhibit COX2 expression ▪ Suppress the NF-κB signaling pathway by stabilizing IκBα and inhibiting its degradation ▪ Inhibit the MAPK signaling cascade, particularly by suppressing MEK and downstream kinases (e.g., JNK, p38) ▪ Inhibit protein tyrosine phosphatases (PTPs) ▪ Promote the activation of the JAK/STAT1 axis ▪ Antidiabetic activity ▪ Neuroprotective activity	[52,53,54,55,56]
	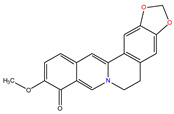 **Berberrubine**	▪ CYP2D6 inactivator ▪ Anticancer activity: inosine monophosphate dehydrogenase inhibitor, thioredoxin reductase inhibitor ▪ Auditory protective effect: promote folate biosynthesis, improve JC-1 signals in HEI-OC1 cells and cochlear hair cells ▪ Promote autophagy in liver macrophages ▪ suppress the STING (stimulator of interferon genes) signaling ▪ Reduce macrophage infiltration and the production of pro-inflammatory cytokines (e.g., IL-6, TNF-α)	[57,58,59,60,61]
	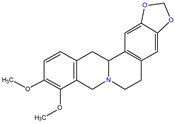 **Canadine**	▪ Antiproliferative and antimetastatic activities	[62,63]
	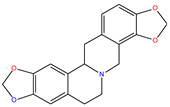 **Stylopine**	▪ Antiviral activity: casein kinase-2 inhibitor ▪ Anticancer activity on osteosarcoma cells	[64,65]
	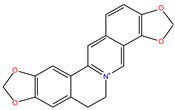 **Coptisine**	▪ Anti-inflammatory activity ▪ Anticancer activity ▪ Antidiabetic activity ▪ Antiviral activity: regulate p21 signaling pathway to inhibit viral replication ▪ Suppress the NLRP3 inflammasome through downregulation expression and decrease levels of cleaved caspase-1, IL-1β, and IL-18 in renal tissue ▪ Decreased serum creatinine and urea nitrogen levels ▪ Downregulate miR-21-5p, a microRNA implicated in fibrogenesis	[66,67,68,69,70]
Protopines	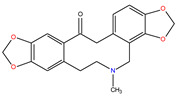 **Protopine**	▪ Reduce inflammatory cell infiltration, decrease levels of pro-inflammatory mediators (e.g., IL-6, IL-13, TNF-α, COX-2, iNOS) ▪ Suppress NLRP3 inflammasome activation ▪ Inhibit the TLR4/MyD88/NF-κB/NLRP3 axis ▪ Anticancer activity by suppressing the PI3K/Akt survival pathway ▪ Antioxidant activity	[71,72,73,74,75]
Aporphine	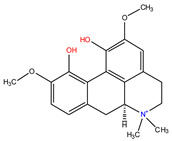 **Magnoflorine**	▪ Neuroprotective activity: inhibit JNK signaling pathway; exhibit an antidepressant effect through the LSD1 target; attenuate the cerebral ischemia-induced neuronal damage ▪ Antidiabetic activity ▪ Anti-inflammatory activity: inhibit the production of nitric oxide, protect the murine macrophage cells from lipopolysaccharide-induced apoptosis	[76,77,78,79,80]
Polyphenols	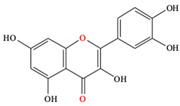 **Quercetin**	▪ Antibacterial, antioxidant, and anticancer activities	[13,81,82]
	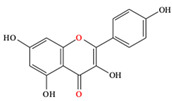 **Kaempferol**	▪ Antioxidant, protective effect against hepatotoxicity	[13,82,83]
	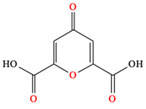 **Chelidonic acid**	▪ Mild analgesic and antimicrobial activities ▪ Regulate levels of interleukin-6 and tumor necrosis factor-α in serum ▪ Attenuate increases in COX-2 and HIF-1α levels	[84,85]
	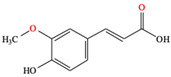 **Ferulic acid**	▪ Antioxidant, immunomodulatory and anti-inflammatory effects	[86]

**Table 2:** Overview of recent experimental bioactivity data for *Chelidonium majus*: plant parts, extraction methods, and biological functions.

Biological Activities	Plant Part	Solvent/Extract Type	Extraction Method	References
Anticancer activity	Aerial part	▪ Ethanol extract	○ Maceration (72 h), ultrasonic extraction, acid–base liquid–liquid extraction	[97]
	Aerial part	▪ Ethanol extract, chloroform alkaloid fraction	○ Maceration (72 h), ultrasonic extraction (5 h), acid–base liquid–liquid extraction	[98]
	Whole plant	▪ Methanol: water: chloroform extraction (molar ratio 2.5:1:1)	○ Solvent extraction, centrifugation, derivatization, vacuum drying	[99]
	Different parts (e.g., leaf, stem, flower, pod and root)	▪ Methanol extract	○ Sequential solvent extraction, centrifugation, vacuum drying	
	Whole plant tissue	▪ Tris-HCl buffer (0.1 M, pH 8.0, with 10% glycerol)	○ Grinding in liquid nitrogen, buffer dissolution, centrifugation for protein extract	[100]
	Milky sap (latex)	▪ Tris-HCl buffer (0.1 M, pH 8.0, with 10% glycerol, 1:2 ratio)	○ Direct sap collection, buffer dissolution, centrifugation for protein fraction	
	Aerial parts	▪ Ethyl acetate ▪ Methanol extract ▪ Ethanol extract	○ Traditional extraction methods (e.g., maceration at room temperature, decoction), solvent evaporation, lyophilization	[101]
	Whole plant	▪ Methanol extract	○ Triple maceration (80% MeOH), acid-base treatment (HCl/NaCl, NaOH), chloroform extraction	[102]
Anti-inflammatory and analgesic activities	Whole plant	▪ Full water extract (FWE) ▪ Protein-enriched fraction (PEF) ▪ Non-protein fraction (NPF)	○ Percolation (3 h, 25 L/min), vacuum concentration, acetone fractionation (4:1) ○ Hot water extraction (90 °C), vacuum concentration, cold acetone precipitation, centrifugation, protein recovery, freeze-drying ○ Hot water extraction (90 °C), vacuum concentration, acetone precipitation (supernatant fraction), freeze-drying	[104]
	Roots and root collars	▪ Methanol extract	○ Ultrasonic-assisted extraction (2 × 2 h), solvent-to-solid 1:20 (*v*/*w*)	[105]
Antiviral activity	Latex	▪ Crude latex (S1) ▪ Protein fraction (S2) ▪ Alkaloid-rich fraction (S3)	○ Centrifugation (14,000 rpm, 20 min, 4 °C); acetone precipitation (1:4 *v*/*v*, −20 °C, overnight); vacuum concentration; PBS resuspension	[107]
Antimicrobial and antifungal activities	Whole plant	▪ Crude 80% MeOH extract ▪ Fractions: n-hexane, EtOAc, BuOH, aqueous	○ Maceration (80% MeOH, 12 h, 25 °C, 200 rpm); evaporation + freeze-drying; partitioning in water with n-hexane, EtOAc, BuOH	[81]
	Whole plant	▪ Total alkaloid extract (80% acidic ethanol, purified via resin adsorption and reflux with dichloromethane:methanol)	○ Reflux extraction (95 °C, 80% acidic ethanol, 1:15, 2 cycles), filtration, evaporation ○ Reflux extraction (DCM:MeOH, 20 h)	[109]
	Aerial and belowground parts	▪ Aqueous extract	○ Ultrasound-assisted extraction (50 °C, 50 min, 360 W, 1:10 ratio)	[111]
Antioxidant activity	Roots, aerial parts	▪ Methanol extract	○ Sonication extraction	[115]
Immunomodulatory activity	Roots and root collars	▪ Methanol extract	○ Ultrasonic-assisted extraction (2 × 2 h), solvent-to-solid 1:20 (*v*/*w*)	[105]
Insecticidal activity	Aerial parts	▪ Hydroalcoholic extract	○ Supersonic extraction	[116]

## Data Availability

Data are contained within the article.

## Abbreviations

APG IV	Angiosperm Phylogeny Group fourth version
CC_50_	Cytotoxic concentration 50%
CDK1	Cyclin-dependent kinase 1
COX2	Cyclooxygenase-2
EGFR	Epidermal growth factor receptor
GPVI	Platelet glycoprotein VI
HEI-OC1	House Ear Institute-Organ of Corti 1
HPLC-DAD	High-performance liquid chromatography with photodiode-array detection
HPV	Human papillomavirus
IL-8	Interleukine-8
JNK	c-Jun N-terminal kinases
LSD1	Lysine-specific demethylase 1A
MBC	Minimum Bactericidal Concentration
MIC	Minimum Inhibitory Concentration
NF-kB	Nuclear factor kappa-light-chain-enhancer of activated B cells
p21, p53	Cyclin-dependent kinase inhibitor
PGE2	Prostaglandin E_2_
SARS-CoV-2	Severe acute respiratory syndrome coronavirus 2
STAT3	Signal transducer and activator of transcription 3
TNF-α	Tumor necrosis factor α
TRPA1	Transient receptor potential ankyrin 1
VEGF	Vascular endothelial growth factor
ZIKV	Zika virus
